# Identification and Characterization of Potential Impurities in Raloxifene Hydrochloride

**DOI:** 10.3797/scipharm.1204-13

**Published:** 2012-05-22

**Authors:** Reguri Buchi Reddy, Thirumani Venkateshwar Goud, Nagabushanam Nagamani, Nutakki Pavan Kumar, Anandan Alagudurai, Raman Murugan, Kannabiran Parthasarathy, Vinayagam Karthikeyan, Perumal Balaji

**Affiliations:** 1 Chemical Research & Development, NPNC Division, Orchid Chemicals and Pharmaceutical Limited, Sozhanganallur, Chennai 600 119, Tamilnadu, India.; 2 Analytical Research & Development, Orchid Chemicals and Pharmaceutical Limited, Sozhanganallur, Chennai 600 119, Tamilnadu, India.

**Keywords:** Raloxifene, Impurities, Isolation, Synthesis, Preparative high performance liquid chromatography, Characterization, Evista

## Abstract

During the synthesis of the bulk drug Raloxifene hydrochloride, eight impurities were observed, four of which were found to be new. All of the impurities were detected using the gradient high performance liquid chromatographic (HPLC) method, whose area percentages ranged from 0.05 to 0.1%. LCMS was performed to identify the mass number of these impurities, and a systematic study was carried out to characterize them. These impurities were synthesized and characterized by spectral data, subjected to co-injection in HPLC, and were found to be matching with the impurities present in the sample. Based on their spectral data (IR, NMR, and Mass), these impurities were characterized as Raloxifene-N-Oxide [Impurity: **1**]; EP impurity A [Impurity: **2**]; EP impurity B [Impurity: **3**]; Raloxifene Dimer [Impurity: **4**]; HABT (6-Acetoxy-2-[4-hydroxyphenyl]-1-benzothiophene or 6-Hydroxy-2-[4-acetoxyphenyl]-1-benzothiophene) [Impurity: **5**]; PEBE (Methyl[4-[2-(piperidin-1-yl)ethoxy]]benzoate) [Impurity: **6**]; HHBA (1-[6-hydroxy-2-(4-hydroxyphenyl)-1-benzothiophen-3-yl]ethanone) [Impurity: **7**]; 7-MARLF (7-Acetyl-[6-hydroxy-2-(4-hydroxyphenyl)-1-benzothiophen-3-yl][4-[2-(piperidin-1-yl)ethoxy]phenyl methanone) [Impurity: **8**]; of which impurities **5–8** are reported for the first time.

## Introduction

Raloxifene, [6-Hydroxy-2-(4-hydroxyphenyl)-1-benzothiophen-3-yl]{4-[2-(piperidin-1-yl)ethoxy]phenyl}methanone hydrochloride, is an estrogen agonist/antagonist, commonly referred to as a selective estrogen receptor modulator (SERM) [1, 2] that belongs to the benzothiophene class of compounds. Raloxifene decreases the resorption of bone and reduces the biochemical markers of bone turnover to the premenopausal range [3–5]. Raloxifene hydrochloride may also lower the chance of developing a certain type of breast cancer (invasive breast cancer) in post-menopausal women [6, 7]. These effects on bone are manifested as reductions in the serum and urine levels of bone turnover markers, decreases in bone resorption based on radiocalcium kinetics studies, increases in bone mineral density [BMD], and decreases in the incidence of fractures. EVISTA (Raloxifene), administered in a 60 mg dose once daily, increased spine and hip BMD by 2 to 3%. EVISTA (Raloxifene) decreased the incidence of the first vertebral fracture from 4.3% for placebo to 1.9%. The presence of impurities in active pharmaceutical ingredients (API) can have a significant impact on the quality and safety of drug products.

**Fig. 1:**
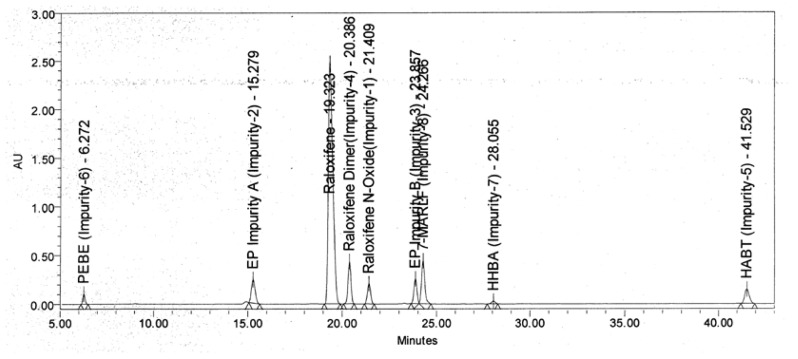
HPLC Chromatogram of Raloxifene Hydrochloride Spiked with impurities

During the analysis of laboratory batches of Raloxifene hydrochloride, eight impurities were observed by the HPLC method (Fig. 1). In order to commercialize an API, it is a mandatory requirement by regulatory authorities to identify and characterize all of the unknown impurities that are present at a level of more than 0.1% [8]. These impurities are required in pure form to check the HPLC method performance in areas such as specificity, linearity, range, accuracy, precision, limit of detection (LOD), limit of quantification (LOQ), robustness, system suitability testing, and relative response factor (RRF) [9]. These related substances are also used to check the accuracy of the analytical method of API.

**Fig. 2:**
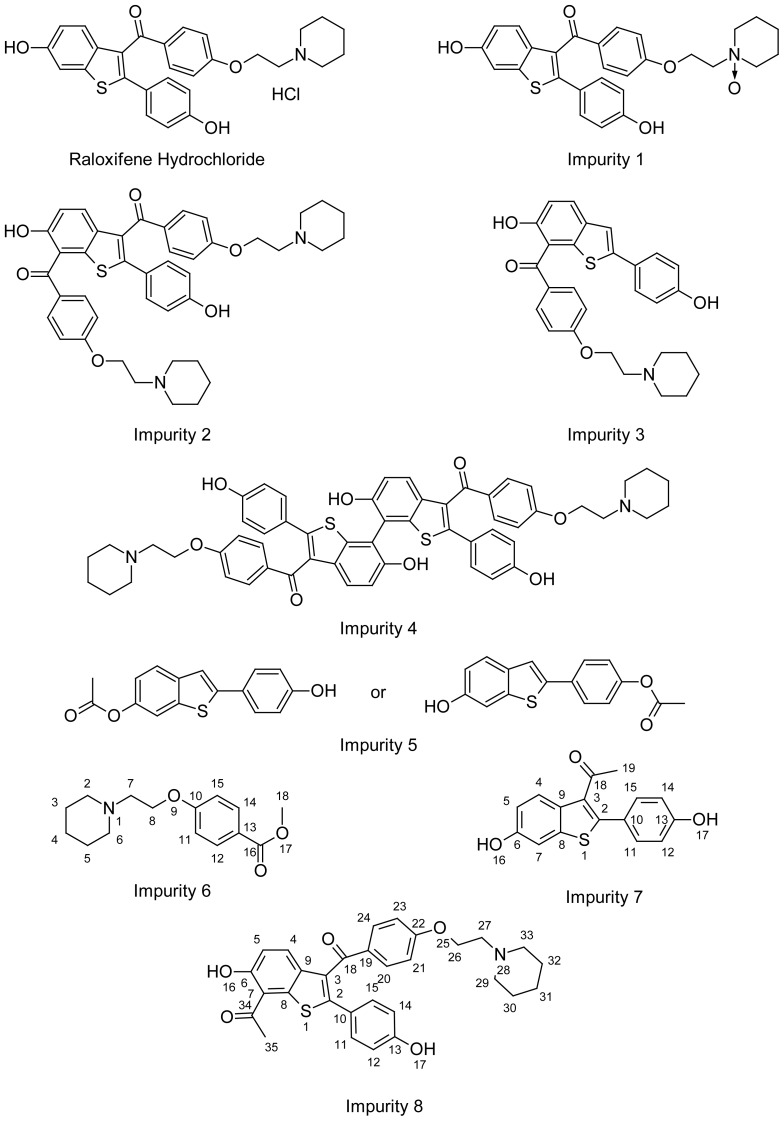
Structures of Raloxifene and impurities **1–8**

**Fig. 3:**
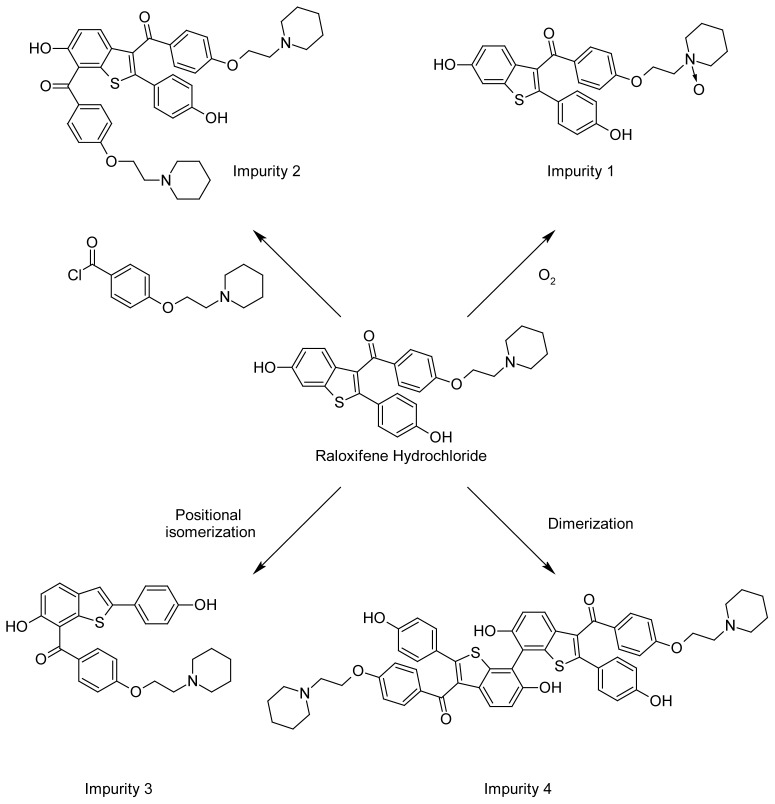
Possible Formation of Impurities

The structure of possible impurities (Fig. 2) related to process/RM/degradants is identified/characterized by the various characterization techniques such as UV, IR, NMR & Mass and chromatographically by HPLC spiking studies. The pathway of the formation of these impurities is also delineated (Fig. 3). Among these eight impurities, impurities **5**–**8** are hitherto not reported, whereas impurities **1**–**4** were reported in the literature [10, 11]. In our manufacturing process of Raloxifene hydrochloride (**I**), we have identified the following eight impurities:

[6-Hydroxy-2-(4-hydroxyphenyl)-1-benzothiophen-3-yl][4-[2-(1-oxidopiperidin-1-yl)ethoxy]phenylmethanone [Impurity: **1**]
[6-Hydroxy-2-(4-hydroxyphenyl)-7-[4-[2-(piperidin-1-yl)ethoxy]benzoyl]-1-benzothiophen-3-yl][4-[2-(piperidin-1-yl)ethoxy]phenylmethanone [Impurity: **2**]
[6-Hydroxy-2-(4-hydroxyphenyl)-1-benzothiophen-7-yl][4-[2-(piperidin-1-yl)ethoxy]phenylmethanone [Impurity: **3**]
[6,6′-Dihydroxy-2,2′-bis(4-hydroxyphenyl)-7,7′-bi-1-benzothiophene-3,3′-diyl]bis{[4-(2-piperidin-1-ylethoxy)phenyl]methanone} [Impurity: **4**]
6-Acetoxy-2-[4-hydroxyphenyl]-1-benzothiophene or 6-Hydroxy-2-[4-acetoxyphenyl]-1-benzothiophene [Impurity: **5**]
Methyl 4-[2-(piperidin-1-yl)ethoxy]benzoate [Impurity: **6**]
1-[6-Hydroxy-2-(4-hydroxyphenyl)-1-benzothiophen-3-yl]ethanone [Impurity: **7**]
7-Acetyl-[6-hydroxy-2-(4-hydroxyphenyl)-1-benzothiophen-3-yl][4-[2-(piperidin-1-yl)ethoxy]phenyl methanone [Impurity: **8**]

Raloxifene impurities **1–3** are reported in the European Pharmacopeia [10]. An increasing number of publications on the synthesis of impurities and the development of analytical methods for bulk drug analysis indicate the significance of impurities of bulk drugs [11–13]. In our present investigation, we have synthesized Impurities **1–4**, and the structural elucidation and characterization by spectral data was carried out on impurities **5–8.**

## Experimental

### Samples and chemicals

Samples of Raloxifene hydrochloride, (Batch No. KGL/RLF-III/1002), were prepared in our R& D laboratories. All eight impurities were synthesized in the laboratory after identification by HPLC and LC-MS. The key starting materials HHBT (2-(4-hydroxyphenyl)-1-benzothiophene-6-ol) and PEBA (4-[2-(piperidin-1-yl)ethoxy]benzoic acid HCl) were purchased from Medilux laboratories Pvt. Ltd. India. HPLC grade acetonitrile and Aluminium trichloride were obtained from Merck, India. *m*-CPBA (*meta*-chloroperoxy-benzoic acid) was purchased from Spectrochem, India. DMAP (4-dimethylaminopyridine) was purchased from Aldrich, India. All other chemicals [potassium dihydrogen orthophosphate, phosphoric acid, potassium hydroxide and hydrogen peroxide (30%) AR grade, sodium thiosulfate, thionyl chloride, triethylamine (TEA), 1,2-dichlorobenzene, acetic anhydride, silicagel (60–120 mesh), sodium bicarbonate, sodium hydroxide, conc. hydrochloric acid, conc. sulfuric acid, methanol, dichloromethane (DCM), dimethyl-formamide (DMF), di-*iso*-propylether (IPE), ethylacetate and *n*-hexane] were obtained from SD Fine chemicals limited, India. Water used for preparing the mobile phase was purified using the Millipore Milli-Q plus (Milford, MA, USA) purification system. Chloroform-*d* and dimethylsulphoxide-*d_6_* were purchased from Euriso-top SA, France.

### High-Performance liquid chromatography (HPLC)

An In-house LC gradient method was developed for the analysis of Raloxifene hydrochloride and its impurities (Water Alliance 2695 separations module & Waters 2487 Dual absorbance detector, with Empower software) using a stainless steel column Inertsil C8-3, (250X4.6) mm, 5μ with a mobile phase consisting of 0.01 M KH_2_PO_4_, pH adjusted to 3.0 (±0.05) with Orthophosphoric acid or potassium hydroxide and acetonitrile in the ratio of 67:33. Composition of eluent was varied at a constant flow rate of 1.0 mL/min and UV detection at 280 nm was used. This LC method was able to separate all of the impurities.

### Liquid chromatography-mass spectrometry (LC-MS)

The mass spectrometry-compatible chromatographic method was developed for the analysis of Raloxifene hydrochloride and its impurities, where a column X-Terra RP-8, (250X4.6) mm was used with a mobile phase consisting of 10mM ammonium formate, pH-adjusted to 3.0 using formic acid and acetonitrile in the gradient method, flow rate of 1.0 ml/min, UV detection at 280 nm, and the column was kept at 45°C. This LC method was able to separate all of the impurities. The mass spectra of impurities were recorded in the API-3000 LC-MS/MS mass spectrometer.

### Mass spectrometry

The LC-MS analysis was performed on the API-3000 LC-MS/MS mass spectrometer [PE Sciex, Foster City, CA]. The analysis was performed in both ionization modes with the Turbo Ion spray interface and the following conditions. For the positive ionization mode, the ion sources used were with a of voltage 5500 V, declustering potential of 10 V, focusing potential of 90 V, entrance potential of 10 V, and nitrogen as the nebulizer gas at 60 psi. In contrast, the negative ionization was performed by switching the polarity of the ion source voltage to 4200 V.

### NMR spectroscopy

The ^1^H NMR and ^13^C NMR experiments for Raloxifene impurities were performed at 400.13 MHz and 100.62 MHz, respectively, on the Bruker Avance 400 MHz FT NMR spectrometer with a multinuclear BBO probe. DMSO-*d_6_* and CDCl_3_ were used as solvents. The ^1^H chemical shift values were reported on the δ scale in ppm, relative to TMS (δ = 0.0 ppm) and in the ^13^C NMR, the chemical shift values were reported relative to CDCl_3_ (δ = 77.0 ppm) and DMSO-*d_6_* (δ = 39.50 ppm) as a reference. The COSY, HSQC and HMBC experiments were performed to assign the signals unequivocally. DEPT-135 spectra revealed the presence of methyl and methine groups as positive peaks and methylene as negative peaks.

### Melting point determination

The melting points of all the impurities were determined by using the capillary method on a POLMON digital melting point apparatus.

### FT-IR spectroscopy

The IR spectra were recorded in the solid state as a KBr dispersion medium using the FT-IR (Perkin Elmer, Spectrum 65 & JASCO-FT-IR-430) spectrophotometer.

### Synthesis of impurities (Fig. 4 and 5)

Impurity **1** is the oxidized product of Raloxifene. During the final stage of Raloxifene synthesis, aerial oxidation leads to the formation of the impurity. This impurity has been prepared in the laboratory via *m*-CPBA oxidation of Raloxifene. Impurity **2** is the side product in the preparation of Raloxifene. Excess of the reagent 4-[2-(piperidin-1-yl)ethoxy]benzoyl chloride (1.4 eq.) used in stage II results in the side product formation. Impurity **3** is also a side product in stage II. Impurity **4** is the dimerized product of Raloxifene. Impurity **5** is formed due to the incomplete acetylation of HHBT (Stage I). Impurity **6** is the side product formed due to the reaction of 4-[2-(piperidin-1-yl)ethoxy]benzoyl chloride with methanol during the conversion of PEBA to PEBA chloride. Impurity **7** is formed through the Fries rearrangement of 6-(acetyloxy)-2-[4-(acetyloxy)phenyl]-1-benzothiophene followed by deprotection. Impurity **8** is a result of Fries rearrangement on acetyl-Raloxifene followed by deprotection.

### Preparation of Impurity 1 [N-Oxide Impurity]

2g of Raloxifene hydrochloride was taken in a mixture of 40ml of DCM, 20ml of methanol and 10ml of 0.5% sodium hydroxide solution. 2.5g of m-CPBA in 20ml of DCM was added slowly to the above reaction mass, followed by dilution with 20ml of methanol, and stirred for 24hrs. The reaction was monitored until completion by HPLC, and then the reaction mass was quenched with 10ml of 1% sodium thiosulphate solution. It was then stirred for one hour. The solid that formed was filtered and washed with water. The final weight was 1.25g.

### Preparation of Impurity 2 [EP Imp A]

20g of PEBA·HCl was taken in 120 ml of DCM, then 0.5ml of DMF and 8.9ml thionyl chloride were added and refluxed for eight hours. Then excess thionyl chloride was distilled out under a vacuum and the residue was dissolved in 300ml of DCM. The charged 29.8g of Raloxifene free base and 14.5ml of TEA were stirred at RT. After reaction completion, it was filtered. The filtrate was washed with water, and the DCM was distilled out under a vacuum. This was added to the mixture containing 750ml 1,2-dichlorobenzene and 35.8g of AlCl_3_. The reaction mass was heated to 125–135 °C for six hours, Then cooled and poured into 2L of 20% HCl solution. The solid was stirred and filtered after that. The wet solid was dissolved in methanol and given carbon treatment. Then the methanol was distilled out completely under a vacuum. The impurity was isolated by preparative HPLC from the above residue

### Preparation of Impurity 3 [EP Imp B]

20g of PEBAHCl was taken in 120ml of DCM, a nd 0.5ml DMF and 8.9 ml thionyl chloride were charged, refluxed for eight hours, and excess thionyl chloride were distilled out under a vacuum. The residue was dissolved in 300 ml of DCM, and 17g of HHBT and 14.5 ml TEA were also charged, and stirred at RT for 12hrs. After reaction completion, the DCM was distilled out under vacuum. The residue was dissolved in 240ml of 1,2-dichloro-benzene and charged 34g of AlCl_3_. It was heated to 140–145 °C for five hours, then cooled and poured into 2L of 20% HCl. Then the solid was filtered, andthe wet solid was dissolved in methanol and given carbon treatment. Then methanol was distilled out under a vacuum. The impurity was isolated by preparative HPLC from the above residue.

### Preparation of Impurity 4 [Dimer]

175g of Raloxifene hydrochloride was taken in 875ml of methanol and it was refluxed for two hours, And cooled to RT and 88ml of conc. hydrochloric acid was charged. It was then stirred for two hours, and the solid was filtered and washed with 350 ml of methanol. The total mother liquor was concentrated and the impurity was isolated by preparative HPLC.

### Preparation of Impurity 5 [HABT]

10 g of 2-(4-hydroxyphenyl)-1-benzothiophene-6-ol and 100 ml of DMF were charged into a 1L RB flask, and added to this was 0.1g of DMAP, which was then cooled to 0–5 °C. 2.5 ml of acetic anhydride was added to the reaction mass at 0–5 °C, and the reaction mass was stirred for one hour at RT and quenched with 500 ml water at 0–5 °C. Then it was stirred for 30 min. and the solid was filtered. The wet cake was washed with 50 ml of IPE and later with 100 ml of water. The solid obtained was purified by silica gel (60–120 Mesh) column chromatography using Hexane and Ethyl acetate (4:6) as eluents.

### Preparation of Impurity 6 [PEBE]

5g of PEBA and 30 ml of methanol were taken in 250 ml RB, and the two ml of conc. Sulfuric acid added to the reaction mass were charged. The reaction mass was heated to reflux and maintained for four hours. The methanol was distilled out and the residue was dissolved in DCM. Then the residue was washed with 10% sodium bicarbonate solution. The organic layer was distilled out completely to obtain the PEBE.

### Preparation of impurity 7 [HHBA]

50 g of 4-[2-(piperidin-4-yl)ethoxy]benzoic acid HCl, 300 ml of DCM and 1.25 ml of DMF were taken in RB, and 20 ml of thionyl chloride at RT was charged and refluxed for eight horrs. Excess thionyl chloride was distilled out, and the residue was dissolved in 750ml DCM and charged with 39g of 4-[6-(acetyloxy)-1-benzothiophen-2-yl]phenyl acetate. The above reaction mixture was charged under stirring into 96.7 g of AlCl_3_ in 250 ml of DCM at RT. After completion, the reaction mass was quenched into 1.2 L of dil. HCl, which was extracted with DCM. The DCM layer was washed with DM water and brine solution. The DCM layer was distilled out completely. The resulting residue was deprotected by being dissolving in 750 ml of methanol and charged with 100 ml of 20% sodium hydroxide solution at RT to obtain Raloxifene free base solution. Methanol was distilled out and charged with 500 ml (1:1) of water–IPE mixture to the residue. The IPE layer was separated from the aq. layer and it was distilled out under a vacuum to get Impurity **7** (1.7 g). Raloxifene (35.0 g) was isolated from the aqueous layer.

### Preparation of Impurity 8 [7-MARLF]

80 g of Raloxifene free base was taken in 2.4L of methanol and charged with 8g of carbon and refluxed for one hour. The solution was cooled and filtered. The filtrate was distilled up to 1.2L. Then 40 ml of conc. HCl in 400 ml of methanol was added to the filtrate. The reaction mixture was stirred for two hours at RT. The resulting salt was filtered and washed with 80ml of methanol to obtain Raloxifene·HCl (70 g). Both of the filtrates were concentrated to obtain a crude mixture which was subjected to preparative HPLC to isolate Impurity 8 (0.2g).

## Results and Discussion

A typical analytical HPLC chromatogram of the laboratory batch of Raloxifene hydrochloride bulk drug recorded using the LC method as described in section 2.2. The target impurities under study are marked as impurity **6** (RT: 22.8, Mass: 263 Da), impurity **7** (RT: 29.7, Mass: 284 Da), impurity **8** (RT: 25.8, Mass: 515 Da). The LC-MS compatible method is used to detect the impurities as described in section 2.3, and to detect all the impurities mentioned in Fig. 1. RRT and name of these impurities and Raloxifene hydrochloride are shown in Table 1. In Table 2 the assigned NMR data of the unknown impurities **6–8** are listed.

### Discussion

Impurity **6** with RRT 0.325, showed *m/z* peaks at 264 [M+H]^+^ with the positive electro spray ionization (ESI) mass. The IR spectrum displayed characteristic absorptions at, 2925, 1585, and 1170 cm^−1^ which is indicative of aromatic –C=C-, -C=O- stretching, and ether functionality. The structure was further supported by quaternary carbon signals in ^13^C and the DEPT-135 spectrum. ^13^C NMR accounted for 15 carbons and the DEPT-135 spectrum displayed seven negative (CH_2_) and five positive which includes one methyl and four aryl groups (CH), and three extra signals appeared in ^13^C other than ^13^C-DEPT, which is considered as three quaternary carbons. Based on the above spectral data, the molecular formula of Impurity **6** is confirmed as C_15_H_21_NO_3_.

Impurity **7** with RRT 1.45, showed *m/z* peaks at 285 [M+H]^+^ with the positive electro spray ionization (ESI) mass. The IR spectrum displayed characteristic absorptions at 3370–3278, 2925, 1654 and 1486–1402 cm^−1^ which is indicative of OH stretching, aliphatic -CH stretching, -C=O stretching, and aromatic –C=C- stretching. The structure was further supported by NMR: ^13^C NMR accounted for 16 carbons, and the DEPT-135 spectrum displayed six positive, which includes one methyl and seven aryl groups [four of them appear as two signals], and eight extra signals appeared in ^13^C other than ^13^C-DEPT, which is considered as eight quaternary carbons. Based on the above spectral data, the molecular formula of Impurity **7** is confirmed as C_16_H_12_O_3_S.

Impurity **8** (7-acetyl-raloxifene) with RRT 1.26, showed *m/z* peaks at 516 [M+H]^+^ with the positive electro spray ionization (ESI) mass. The IR spectrum displayed characteristic absorptions at 3435, 2927–2855, 1639-1598, and 1536-1506 cm,^−1^ which is indicative of -OH stretching, -CH stretching, -C=O stretching, and C=C- aromatic stretching. The structure was further supported by NMR: ^13^C NMR accounted for 30 carbons, and the DEPT-135 spectrum displayed five negative (CH_2_) and seven positive, which includes one methyl, seven methylene groups [four of them appear as two signals], and 10 aromatic carbons [eight of them appear as four signals]. Eleven extra signals appeared in ^13^C other than the ^13^C-DEPT, which is considered as twelve quaternary carbons [one quaternary carbon merged with an aromatic carbon signal]. Based on the above spectral data, the molecular formula of Impurity **8** is confirmed as C_30_H_29_NO_5_S.

## Conclusion

Information about the various possible impurities, metabolites, and their synthetic routes is a prerequisite for the thorough understanding of impurity profiles in the manufacturing of the selective estrogen receptor modulator drug, Raloxifene. Keeping in view, this regulatory requirement of Raloxifene impurities, the process-related impurities, and metabolites in Raloxifene bulk drug were identified, synthesized, and characterized using mass, IR, and NMR spectral data.

## Figures and Tables

**Fig. 4:**
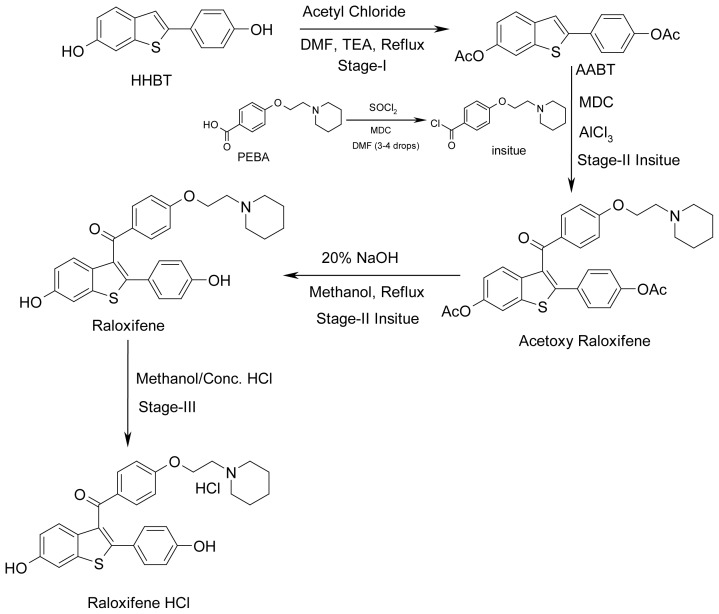
Scheme for the synthesis of Raloxifene

**Fig. 5:**
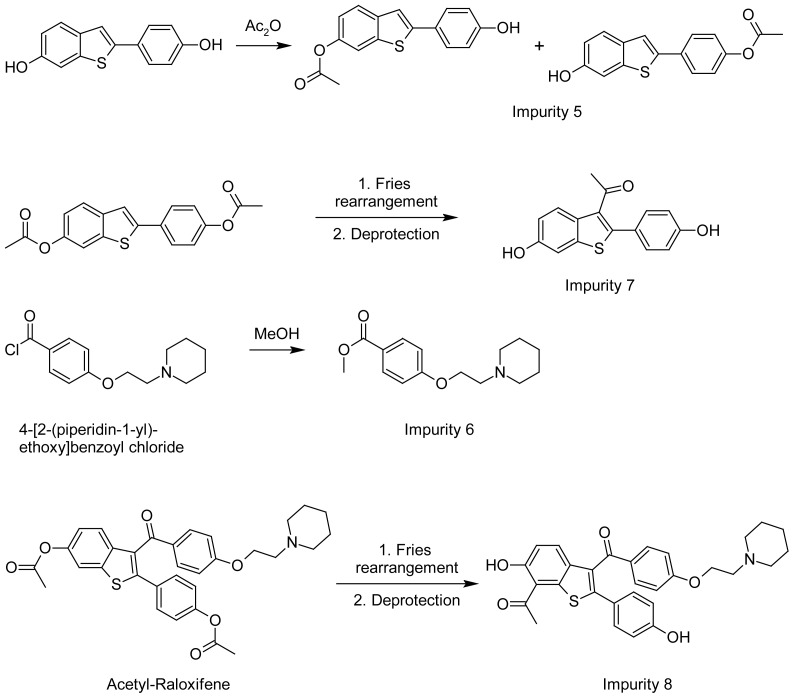
Scheme for the synthesis of Impurities

**Tab. 1:** Relative retention time of known eluting peaks with respect to Raloxifene peaks are given below

Sl.No	Name of impurity	RRT
1	PEBE [Impurity 6]	0.325
2	EP impurity A [Impurity 2]	0.791
3	Raloxifene	1.0
4	Raloxifene Dimer [Impurity 4]	1.055
5	Raloxifene-N-Oxide [Impurity 1]	1.108
6	EP impurity B [Impurity 3]	1.235
7	7-MARLF [Impurity 8]	1.256
8	HHBA [Impurity 7]	1.452
9	HABT [Impurity 5]	2.149

**Tab. 2:** ^1^H and ^13^C NMR assignment for Impurities **6–8**

Atom Nr.	Impurity 6				Impurity 7				Impurity 8			
		
	^1^H	ppm/J	^13^C	DEPT	^1^H	ppm/J	^13^C	DEPT	^1^H	ppm/J	^13^C	DEPT
1												
2	2	2.42/m	54.4	-CH2			146.3				143.9	
3	2	1.46–1.52/m	25.6	-CH2			130.8				129.8	
4	2	1.37/m	23.9	-CH2	1	7.86/d, 8.8	124.7	-CH	1	7.53/d, 8.7	128.6	-CH
5	2	1.46–1.52/m	25.6	-CH2	1	6.92/dd, 8.9,2.2	115.5	-CH	1	7.09/d, 8.6	117.1	-CH
6	2	2.42/m	54.4	-CH2			155.4				159.4	
7	2	2.64/t,5.8	57.2	-CH2	1	7.28/d,2.2	106.7	-CH			117.2	
8	2	4.12/t,5.8	65.9	-CH2			139.1				138.7	
9							131.6				132.5	
10			162.4				123.9				123.9	
11	1	7.03/d,8.7	114.5	-CH	1	6.89/d,8.5	130.7	-CH	1	7.18/d,8.6	129.7	-CH
12	1	7.89/d,8.7	131.2	-CH	1	7.3/d,8.5	115.9	-CH	1	6.67/d,8.6	115.7	-CH
13			121.7				158.7				157.8	
14	1	7.89/d,8.7	131.2	-CH	1	7.3/d,8.5	115.9	-CH	1	6.67/d,8.6	115.7	-CH
15	1	7.03/d,8.7	114.5	-CH	1	6.89/d,8.5	130.7	-CH	1	7.18/d,8.6	129.7	-CH
16			165.9	-C=O	1	9.77/s		-OH				
17					1	9.96/s		-OH	1	9.72/s		-OH
18	3	3.82,s	51.8	-CH3			197.5	-C=O			192.9	-C=O
19					3	2.08/s,	30.8	-CH_3_			129.7	
20									1	7.61d,8.8	131.8	-CH
21									1	6.89/d,8.8	114.5	-CH
22											162.8	
23									1	6.89/d,8.8	114.5	-CH
24									1	7.61d,8.8	131.8	-CH
25												
26									2	4.05,t,5.8	65.9	-CH2
27									2	2.61/t,5.8	57.1	-CH2
28												
29									2	2.39/m	54.3	-CH2
30									2	1.36–1.48/m	25.5	-CH2
31									2	1.35/m	23.8	-CH2
32									2	1.36–1.48/m	25.5	-CH2
33									2	2.39/m	54.3	-CH2
34											196.7	-C=O
35									3	2.75/s	32.5	-CH3

The D_2_O exchange study confirmed the presence of one exchangeable proton in impurity **8** other than –OH proton which is not observed in the ^1^H NMR spectrum and two exchangeable protons in impurity **7**.
